# Analyzing the impact of glycemic metabolic status on cardiovascular mortality and all-cause mortality related to the estimated glucose disposal rate: a nationwide cohort study

**DOI:** 10.3389/fendo.2024.1494820

**Published:** 2025-01-21

**Authors:** Shiming He, Chao Wang, Xin Huang, Guoan Jian, Zihao Lu, Kun Jiang, Guobo Xie, Guotai Sheng, Yang Zou

**Affiliations:** ^1^ Jiangxi Medical College, Nanchang University, Nanchang, Jiangxi, China; ^2^ Jiangxi Provincial Geriatric Hospital, Jiangxi Provincial People’s Hospital, The First Affiliated Hospital of Nanchang Medical College, Nanchang, Jiangxi, China; ^3^ Jiangxi Cardiovascular Research Institute, Jiangxi Provincial People’s Hospital, The First Affiliated Hospital of Nanchang Medical College, Nanchang, Jiangxi, China

**Keywords:** insulin resistance, estimated glucose disposal rate, cardiovascular mortality, eGDR, all-cause mortality

## Abstract

**Objective:**

The Estimated Glucose Disposal Rate (eGDR) serves as a surrogate marker for insulin resistance, with numerous studies highlighting its significant prognostic value. This paper aims to analyze the impact of eGDR on cardiovascular and all-cause mortality across different glycemic metabolic statuses, including normal fasting glucose (NFG), prediabetes, and diabetes.

**Methods:**

This study included 46,016 American adults who underwent health examinations as part of the National Health and Nutrition Examination Survey from 1999 to 2018. Multivariable Cox regression was employed to explore the relationships between eGDR and mortality rates under varying glycemic states. Additionally, Kaplan-Meier curves were used to compare the cumulative incidence of cardiovascular and all-cause mortality across different metabolic statuses. Finally, the predictive value of eGDR for mortality was assessed using receiver operating characteristic curves.

**Results:**

During an average follow-up of 115 months, a total of 6,906 (15.01%) participants experienced all-cause mortality, with 1,798 (3.91%) deaths attributed to cardiovascular causes. Kaplan-Meier analysis revealed that higher eGDR levels were associated with gradually reduced mortality rates. After adjusting for confounders, elevated eGDR levels were protective against both cardiovascular and all-cause mortality; the protective effect was notably stronger for cardiovascular mortality [Cardiovascular mortality hazard ratio: 0.92; All-cause mortality hazard ratio: 0.94]. Further interaction tests indicated that glycemic status significantly modified the protective effect of eGDR (*P*-interaction<0.0001); specifically, high eGDR conferred stronger protection against cardiovascular and all-cause mortality in individuals with NFG and prediabetes compared to those with diabetes. Receiver operating characteristic analysis suggested that eGDR had superior predictive value for mortality in the NFG and prediabetic populations compared to the diabetic group.

**Conclusion:**

eGDR is a straightforward surrogate for insulin resistance, acting as a protective factor against cardiovascular and all-cause mortality in American adults, with glycemic status modifying this protective effect. Specifically, high eGDR levels offer stronger protection in individuals with NFG and prediabetes compared to those with diabetes; moreover, eGDR appears to be more suitable for predicting mortality events in the NFG and prediabetic populations.

## Background

Cardiovascular diseases (CVD) are a major health concern worldwide (1). According to the Global Burden of Disease study, the total incidence of CVD increased from 271 million cases in 1990 to 523 million cases in 2019, with associated cardiovascular deaths rising from 12.1 million in 1990 to 18.6 million in 2019 (1, 2). Despite intensified efforts in primary and secondary prevention and the widespread dissemination of knowledge about cardiovascular health, emphasizing the importance of lifestyle changes (3, 4), the global incidence of CVD continues to rise significantly (1). This suggests that there may be unrecognized residual cardiovascular risks (5). Current research largely agrees that adverse metabolic factors such as dyslipidemia (6, 7), hypertension (8), and abnormal glucose metabolism (9) are closely linked to the onset of CVD, with hypertension showing the strongest correlation (10).

Insulin resistance (IR) is a metabolic condition characterized by reduced responsiveness of target organs or tissues to insulin (11), leading to dysregulation of blood glucose (12) and affecting overall metabolic health. Extensive research has confirmed that IR increases the risk of stroke (12), CVD (11–13), and mortality (14–17); thus, understanding the characteristics and effects of IR is crucial for the prevention and treatment of metabolic diseases. Against this backdrop, several methods for assessing IR have been developed: (i) direct methods such as the hyperinsulinemic-euglycemic clamp technique and the insulin suppression test (18), which are invasive and costly, limiting their clinical application. (ii) Simple surrogate markers for IR, such as the Homeostatic Model Assessment for IR (HOMA-IR) (19), the triglyceride-glucose index (20), and the Estimated Glucose Disposal Rate (eGDR) (21). Previous studies predominantly used HOMA-IR to define IR; however, HOMA-IR is susceptible to interference from medications such as insulin, insulin sensitizers, and insulin secretagogues (22, 23). Furthermore, the newly developed triglyceride-glucose index, although it includes triglycerides (TG) (20), does not account for hypertension, which may weaken its effectiveness in assessing CVD risk. The eGDR is a new surrogate marker calculated based on obesity index waist circumference (WC), glycemic index glycated hemoglobin (HbA1c), and hypertension; studies suggest that eGDR has a similar accuracy to the hyperinsulinemic-euglycemic clamp technique in assessing IR status (24). Moreover, clinical evidence indicates that a lower eGDR is associated with an increased risk of mortality from various diseases (25–30); however, these studies primarily focus on individuals with diabetes or prediabetes, which could exaggerate or obscure the role of eGDR, necessitating further clarification of the impact of glycemic metabolic status on eGDR-related mortality. To address this issue, the present study aims to assess the impact of eGDR on cardiovascular and all-cause mortality among the US population across different glycemic metabolic statuses using follow-up data from the National Health and Nutrition Examination Survey (NHANES) 1999-2018.

## Methods

### Data source

The data used in this analysis is publicly available through the NHANES database. NHANES, conducted by the National Center for Health Statistics (NCHS) of the Centers for Disease Control and Prevention, is an extensive health survey that includes a broad range of health and nutritional information of the U.S. population. This study employed a complex, multistage, stratified sampling design and received approval from the NCHS Research Ethics Review Board. All NHANES participants provided informed consent. As this study utilized de-identified public data, it was exempt from Institutional Review Board approval.

**Figure 1:**
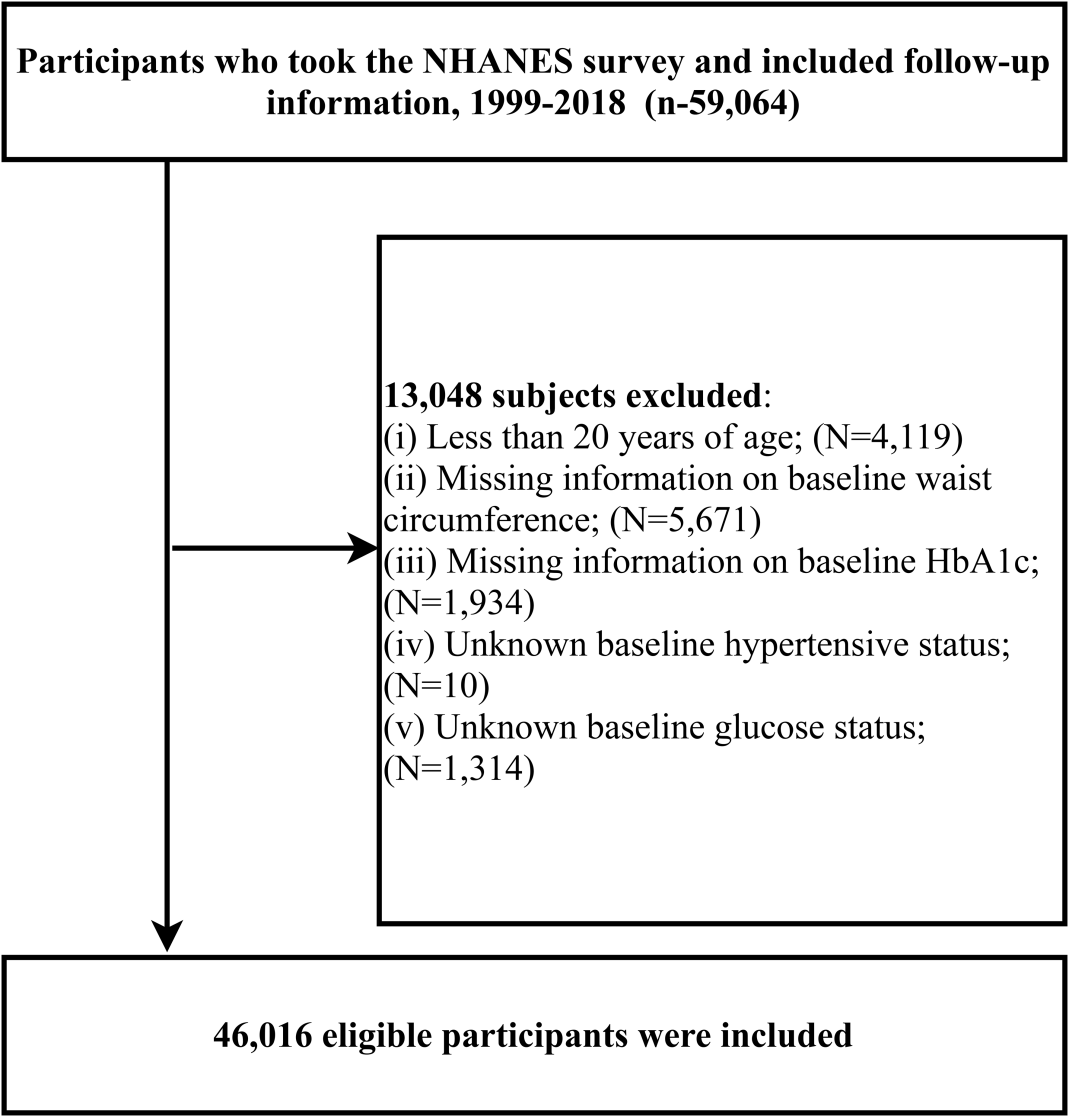
Study population inclusion flow chart.

For the exploration of the impact of eGDR on cardiovascular and all-cause mortality rates among the U.S. population, we included 59,364 participants registered at NHANES mobile examination centers from 1999 to 2018 with subsequent follow-up information, extracting demographic, clinical examination, laboratory data, and survey data. Participants were excluded if they were: (1) under the age of 20 (n=4,419); (2) missing baseline WC information (n=5,671) or baseline HbA1c (n=1,934); (3) uncertain hypertension status at baseline (n=10); (4) uncertain glycemic metabolic status (n=1,314). A total of 46,016 participants were finally included in the study related to eGDR. The inclusion and exclusion processes were detailed in **Figure 1**.

### Data collection and processing

First, we extracted general demographic information from the NHANES database’s demographic and questionnaire modules, such as age, gender, race, poverty income ratio, educational level, disease information [hypertension, congestive heart failure, coronary heart disease (CHD)], disease treatment information (antihypertensive therapy, hypoglycemic therapy, lipid-lowering therapy) and smoking/drinking status, which were obtained using standardized questionnaires by trained survey personnel. Educational levels were categorized as Less than 9th grade, 9-11th grade, High School Grad/General Educational Development or Equivalent, and College graduate or above. Smoking and drinking status were divided into three categories: never, former, and current (31).

From the NHANES laboratory data module, we retrieved relevant biochemical metabolic markers, including total cholesterol (TC), TG, high-density lipoprotein cholesterol (HDL-C), low-density lipoprotein cholesterol (LDL-C), alanine aminotransferase (ALT), aspartate aminotransferase (AST), uric acid (UA), estimated glomerular filtration rate (eGFR), and fasting plasma glucose (FPG).

Important anthropometric measurements such as weight, height, WC, systolic blood pressure (SBP), and diastolic blood pressure (DBP) were extracted from the NHANES examination data module. All fasting biochemical tests and anthropometric measurements were conducted according to standardized procedures in mobile examination centers or corresponding laboratories. Hypertension diagnosis was based on the 2022 American clinical guidelines for hypertension, defined as an average SBP≥130 mmHg or DBP≥80 mmHg, or current use of antihypertensive medication (32).

Glycemic metabolic status was defined according to the American Diabetes Association guidelines as normal fasting glucose (NFG), prediabetes, and diabetes (33), where diabetes includes self-reported diagnosis, use of insulin or oral hypoglycemic agents, FPG≥126 mg/dL or HbA1c level≥6.5%; prediabetes is diagnosed based on self-reported prediabetes or FPG between 100 mg/dL and 125 mg/dL or HbA1c between 5.7% and 6.4%; NFG is defined as FPG <100 mg/dL or HbA1c <5.7%.

### Mortality ascertainment

Mortality data were obtained from death certificate records in the National Death Index (provided by NCHS), updated until December 31, 2019. The primary outcomes considered in our study included CVD-specific mortality and all-cause mortality (34).

### eGDR calculation formula

The formula for calculating eGDR is as follows: eGDR = 21.158 − (0.09*WC) − (3.407*hypertension) − (0.551*HbA1c), where WC is in centimeters (cm), hypertension is coded as no=0/yes=1, and HbA1c is in percentage (%) (21).

### Statistical analysis

In this study, statistical analyses were performed using Empower(R) version 4.2 and R language version 4.2.1. Consistent with NHANES data analysis guidelines, all analyses accounted for sample weights (35). In the baseline characteristics description, the study population was divided into four groups according to eGDR quartiles. Continuous variables were presented as survey-weighted means [95% confidence interval (CI)] and categorical variables as unweighted frequencies (weighted percentages); group differences were assessed using survey-weighted linear regression and survey-weighted Chi-square tests.

Before examining the impact of eGDR on cardiovascular and all-cause mortality rates among individuals with different glycemic metabolic statuses, we calculated the variance inflation factor for all variables using multiple linear regression equations and excluded any variables with a variance inflation factor≥5 from subsequent analyses (**Supplementary Tables S1**, **S2**) (36). Following the STROBE guidelines, we evaluated three progressively adjusted mult-class logistic regression models to analyze the association between eGDR and prediabetes and diabetes; Additionally, we also constructed three multivariable Cox proportional hazards regression models to explore the effects of eGDR on cardiovascular and all-cause mortality among the U.S. population, testing the proportional hazards assumption using Schoenfeld residuals. Model I adjusted for key demographic indicators (age, gender, race), poverty-income ratio, and education; Model II further adjusted for simple anthropometric parameters and laboratory biochemical markers (height, BMI, SBP, DBP, ALT, AST, HDL-C, eGFR, HbA1c, UA); Model III further adjusted for lifestyle habits and medical history [drinking status, smoking status, congestive heart failure, CHD]; Model IV, as the final model, included additional adjustments for disease treatment information (antihypertensive therapy, hypoglycemic therapy, lipid-lowering therapy) (37, 38).

After establishing an independent association between eGDR and mortality, we further assessed the differences in the association between eGDR and mortality rates across different glycemic metabolic statuses based on Model IV. Additionally, Kaplan-Meier survival curves were plotted to illustrate the cumulative incidence of cardiovascular and all-cause mortality across different levels of eGDR and glycemic statuses, with statistical differences evaluated using the log-rank test.

To visually observe the association between eGDR and mortality rates, a four-knot restricted cubic spline (RCS) was constructed based on Model IV to evaluate the impact of eGDR on cardiovascular and all-cause mortality rates across different glycemic metabolic statuses. Upon detecting potential nonlinear associations, segmented Cox regression was applied using a recursive algorithm to calculate the optimal threshold values for eGDR.

Finally, receiver operating characteristic (ROC) curves were plotted to predict cardiovascular and all-cause mortality rates among different glycemic metabolic statuses based on eGDR, with the area under the curve (AUC) calculated accordingly. All *P*-values were two-tailed, with significance set at *P* < 0.05.

## Results

### Baseline characteristics

In this study, a total of 46,016 participants were included, comprising 23,162 males and 22,854 females, with an average age of 50 years. Baseline characteristics summarized by eGDR quartiles (**Table 1**, Q1: ≤5.21; Q2: 5.21-7.05; Q3: 7.05-9.71; Q4: ≥9.71) revealed that compared to participants in the lowest quartile (Q1), those in the highest quartile (Q4) tended to be younger, had lower BMI, more optimal heights and weights, better blood pressure levels, and biochemical markers closer to normal ranges (including HDL-C, LDL-C, TC, TG, ALT, AST, eGFR, HbA1c, FPG, UA). Additionally, compared to Q1, there was a gradual decrease in the proportion of male participants and an increase in female participants in Q4; those in Q4 also had higher educational levels, were more likely to smoke and drink, had a lower proportion of antihypertensive, hypoglycemic, and lipid-lowering therapies, and fewer instances of congestive heart failure and CHD.

**Table 1:** Summary of study population baseline characteristics according to eGDR quartiles.

	eGDR quartiles				*P*-value
	Q1 (≤5.21)	Q2 (5.21-7.05)	Q3 (7.05-9.71)	Q4 (≥9.71)	
Age, years	54.96 (54.53,55.40)	53.53 (53.08,53.98)	45.66 (45.20,46.13)	37.22 (36.79,37.64)	<0.0001
PIR	2.95 (2.88,3.01)	3.07 (3.00,3.13)	3.00 (2.93,3.06)	3.06 (2.99,3.13)	0.0004
Weight, kg	103.47 (102.87,104.07)	80.90 (80.47,81.33)	81.73 (81.26,82.19)	66.42 (66.18,66.67)	<0.0001
Height, cm	170.32 (170.00,170.63)	168.55 (168.28,168.82)	169.03 (168.76,169.30)	167.51 (167.26,167.75)	<0.0001
BMI, kg/m^2^	35.69 (35.48,35.89)	28.45 (28.31,28.59)	28.53 (28.39,28.67)	23.64 (23.56,23.73)	<0.0001
WC, cm	117.63 (117.25,118.01)	98.89 (98.61,99.16)	97.96 (97.60,98.32)	83.32 (83.12,83.52)	<0.0001
HDL-C, mmol/L	1.21 (1.20,1.22)	1.37 (1.36,1.38)	1.36 (1.35,1.37)	1.51 (1.50,1.52)	<0.0001
LDL-C, mmol/L	2.96 (2.93,3.00)	3.14 (3.10,3.17)	3.10 (3.07,3.13)	2.83 (2.80,2.86)	<0.0001
TC, mmol/L	5.09 (5.06,5.13)	5.30 (5.27,5.34)	5.14 (5.11,5.16)	4.83 (4.80,4.85)	<0.0001
TG, mmol/L	1.90 (1.84,1.97)	1.65 (1.59,1.71)	1.49 (1.45,1.53)	1.06 (1.04,1.08)	<0.0001
ALT, U/L	29.47 (28.90,30.05)	26.55 (26.09,27.00)	26.02 (25.35,26.69)	21.57 (21.23,21.92)	<0.0001
AST, U/L	26.44 (26.05,26.84)	26.21 (25.77,26.65)	24.97 (24.65,25.29)	23.66 (23.36,23.95)	<0.0001
eGFR, mL/min/1.73 m^2^	86.85 (86.26,87.44)	87.80 (87.21,88.39)	95.88 (95.27,96.49)	102.96 (102.32,103.60)	<0.0001
HBA1c, %	6.25 (6.21,6.29)	5.56 (5.54,5.59)	5.44 (5.42,5.45)	5.19 (5.18,5.20)	<0.0001
FPG, mmol/L	7.01 (6.93,7.10)	5.82 (5.77,5.86)	5.59 (5.56,5.63)	5.20 (5.18,5.22)	<0.0001
UA, ummol/L	361.52 (359.09,363.95)	335.12 (332.95,337.28)	318.88 (316.80,320.96)	283.77 (282.07,285.46)	<0.0001
SBP, mmHg	132.28 (131.81,132.75)	131.33 (130.86,131.80)	118.74 (118.32,119.15)	110.10 (109.84,110.36)	<0.0001
DBP, mmHg	74.86 (74.40,75.32)	75.22 (74.80,75.64)	70.07 (69.74,70.40)	66.38 (66.09,66.66)	<0.0001
Gender					<0.0001
Female	43.48 (42.19,44.78)	47.36 (46.11,48.60)	49.70 (48.55,50.85)	61.21 (60.02,62.38)	
Male	56.52 (55.22,57.81)	52.64 (51.40,53.89)	50.30 (49.15,51.45)	38.79 (37.62,39.98)	
Race					<0.0001
Other Hispanic	4.46 (3.67,5.40)	5.00 (4.18,5.98)	5.73 (4.85,6.75)	6.95 (5.92,8.14)	
Non-Hispanic Whit	70.38 (67.92,72.73)	70.68 (68.47,72.79)	68.24 (65.92,70.47)	67.12 (65.03,69.14)	
Non-Hispanic Black	13.54 (12.00,15.24)	10.76 (9.64,11.99)	9.77 (8.74,10.92)	8.75 (7.89,9.71)	
Mexican American	7.13 (5.94,8.52)	6.59 (5.70,7.61)	9.88 (8.61,11.32)	8.55 (7.60,9.60)	
Other Race	4.50 (3.88,5.21)	6.97 (6.19,7.84)	6.37 (5.64,7.19)	8.64 (7.80,9.56)	
Education					<0.0001
Less than 9th grade	6.78 (6.11,7.52)	6.92 (6.27,7.62)	5.76 (5.24,6.34)	4.39 (3.92,4.91)	
9-11th grade	12.34 (11.49,13.25)	11.58 (10.80,12.41)	11.50 (10.69,12.37)	9.74 (8.90,10.66)	
High School Grad/GED or Equivalent	26.77 (25.56,28.02)	25.51 (24.20,26.87)	25.07 (23.96,26.21)	19.92 (18.81,21.08)	
College graduate or above	54.11 (52.57,55.64)	55.99 (54.26,57.70)	57.67 (56.06,59.26)	65.95 (64.07,67.77)	
Drinking status					<0.0001
Never	11.47 (10.52,12.50)	11.76 (10.86,12.73)	10.62 (9.55,11.78)	10.84 (9.73,12.07)	
Former	21.27 (19.88,22.73)	15.66 (14.57,16.81)	12.93 (12.07,13.84)	8.90 (8.17,9.69)	
Current	67.26 (65.41,69.05)	72.58 (70.97,74.14)	76.45 (74.97,77.88)	80.26 (78.70,81.74)	
Smoking status					<0.0001
Never	48.41 (47.01,49.81)	52.37 (51.03,53.71)	52.40 (50.95,53.84)	59.30 (57.77,60.81)	
Former	34.55 (33.34,35.79)	27.40 (26.20,28.64)	22.94 (21.82,24.11)	17.04 (16.01,18.13)	
Current	17.04 (16.17,17.94)	20.22 (19.20,21.29)	24.66 (23.47,25.90)	23.66 (22.32,25.04)	
Congestive heart failure	753 (6.58%)	413 (3.60%)	199 (1.73%)	48 (0.42%)	<0.0001
Coronary heart disease	908 (7.95%)	634 (5.54%)	297 (2.59%)	76 (0.66%)	<0.0001
Antihypertensive therapy	7005 (60.96%)	5072 (44.13%)	1718 (14.94%)	297 (2.58%)	<0.0001
Hypoglycemic therapy	3898 (33.92%)	2718 (23.65%)	1346 (11.71%)	330 (2.87%)	<0.0001
Lipid-lowering therapy	3355 (29.19%)	966 (8.41%)	537 (4.67%)	68 (0.59%)	<0.0001

PIR, Poverty income ratio; BMI, body mass index; WC, Waist circumference; ALT, alanine aminotransferase; AST, aspartate aminotransferase; HDL-C, high-density lipoprotein cholesterol; TC, total cholesterol; TG, triglyceride; LDL-C, low density lipoprotein cholesterol; HbA1c, hemoglobin A1c; FPG, fasting plasma glucose; UA, uric acid; eGFR, estimated glomerular filtration rate; SBP, systolic blood pressure; DBP, Diastolic blood pressure; eGDR, estimated glucose disposal rate.

For continuous variables: survey-weighted mean (95% CI), P-value was by survey-weighted linear regression (svyglm); For categorical variables: survey-weighted percentage (95% CI), P-value was by survey-weighted Chi-square test (svytable).

### eGDR association with prediabetes and diabetes

Based on cross-sectional survey data, we analyzed the association of eGDR with prediabetes and diabetes through multi-class logistic regression. The results of the study showed that after fully adjusting for confounders, eGDR was negatively associated with both prediabetes and diabetes (**Supplementary Table S3**); however, this negative association with diabetes was comparatively weaker.

### Follow-up results

During an average follow-up of 115 months, 6,906 participants (15.01%) experienced all-cause mortality, of which 1,798 deaths (3.91%) were attributed to cardiovascular causes. Compared to the lowest eGDR quartile (Q1), higher eGDR quartiles showed a gradual decrease in the proportion of cardiovascular and all-cause mortality (**Supplementary Table S4**). Further stratification by glycemic metabolic status revealed that individuals with diabetes had higher proportions of cardiovascular and all-cause mortality compared to those with NFG and prediabetes (**Supplementary Table S5**).

**Figure 2:**
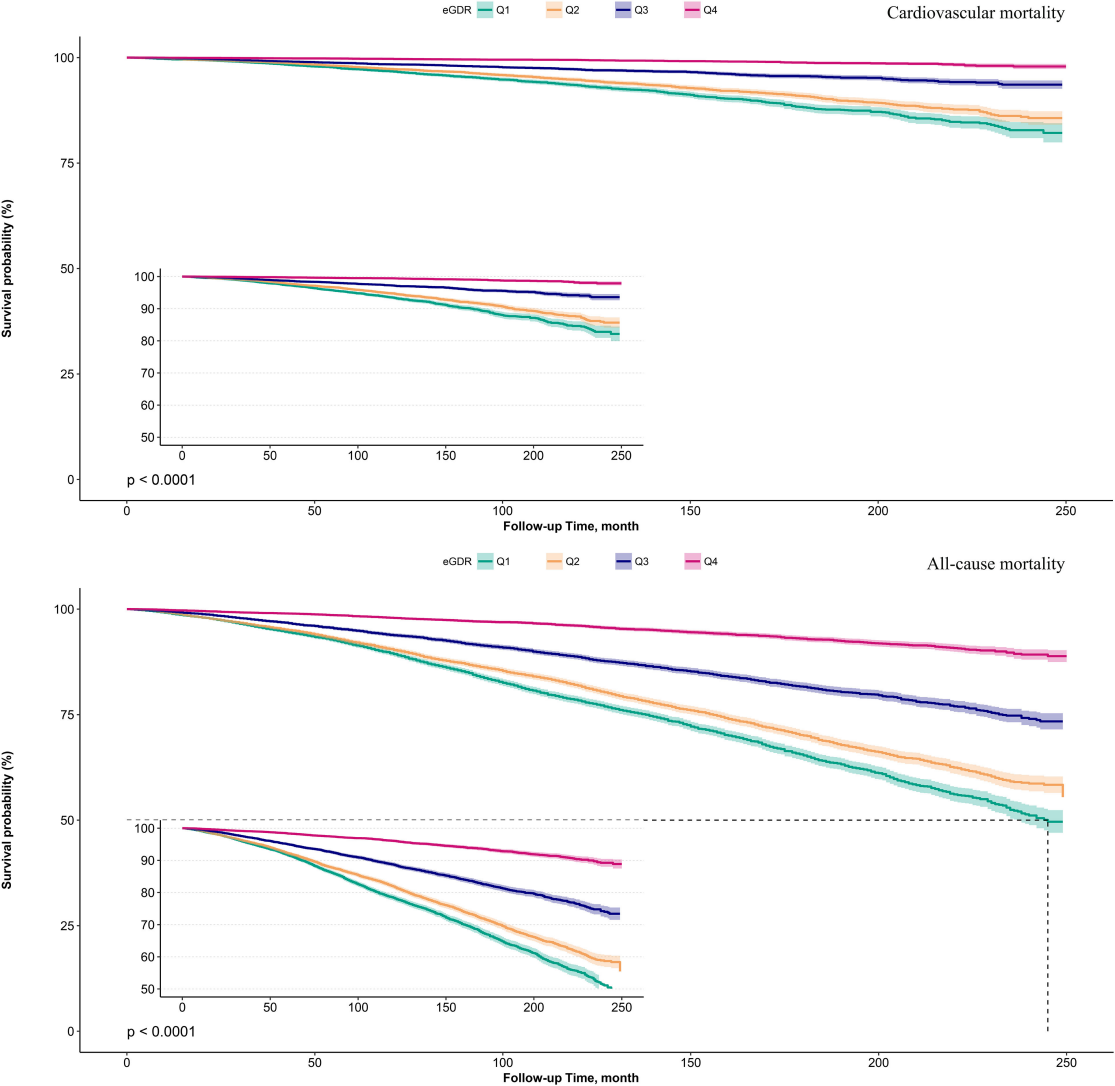
Survival curves showing cardiovascular mortality and all-cause mortality in the study population according to eGDR quartiles.

**Figure 3:**
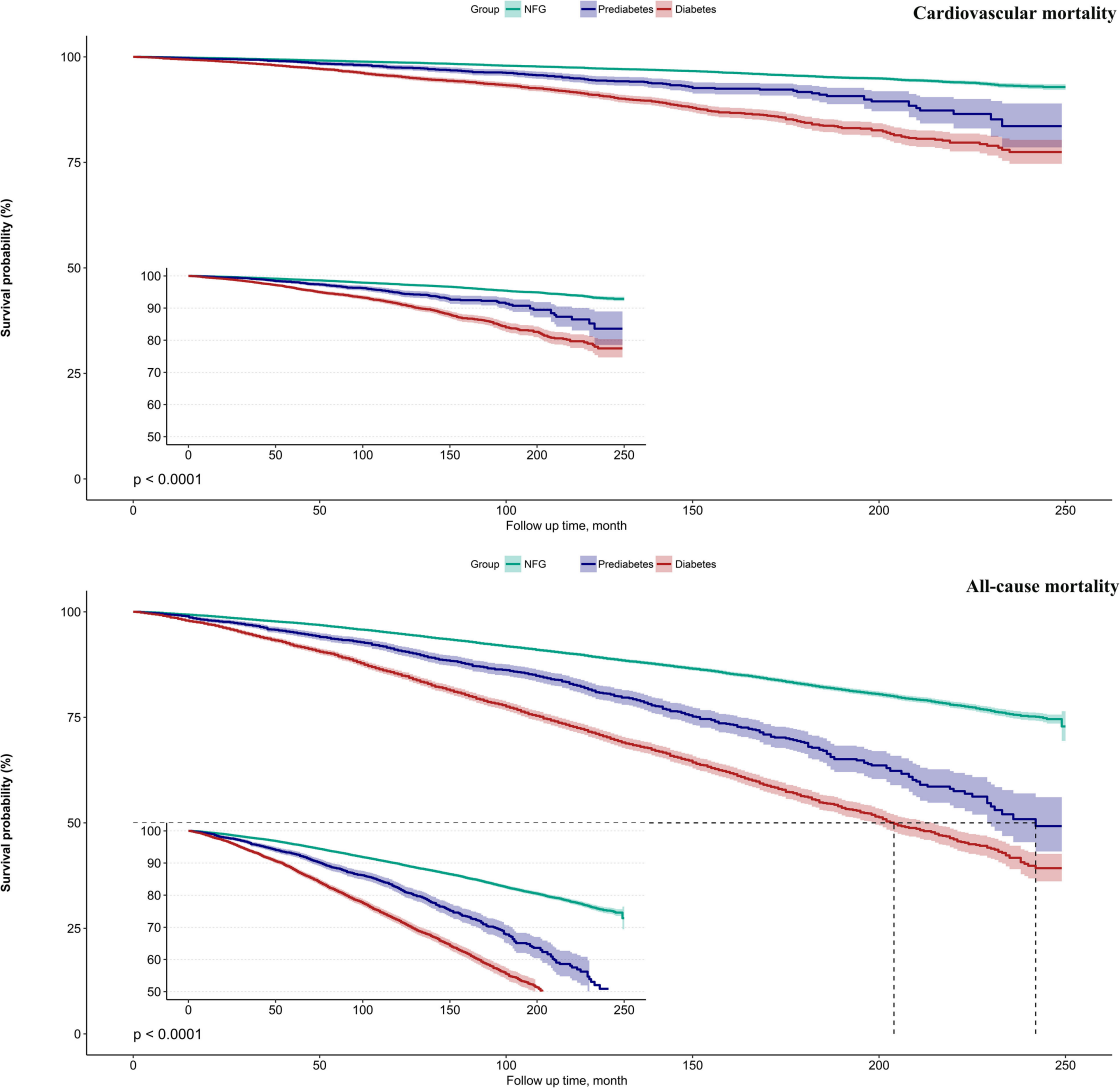
Survival curves showing cardiovascular mortality and all-cause mortality in the study population according to glycemic metabolic status.

Kaplan-Meier survival curves illustrated by eGDR quartiles (**Figure 2**) demonstrated that as eGDR quartiles increased, the risks of cardiovascular and all-cause mortality progressively decreased. When stratified by glycemic metabolic status, those with NFG and prediabetes exhibited relatively lower risks of cardiovascular and all-cause mortality compared to individuals with diabetes (**Figure 3**).

### Independent association between eGDR and mortality

**Table 2** presents the associations between baseline eGDR and cardiovascular and all-cause mortality across three progressively adjusted Cox regression models. A consistent negative correlation was observed between eGDR and both cardiovascular and all-cause mortality across Models I-IV. In the fully adjusted model (Model IV), each unit increase in eGDR was associated with a 8% reduction in cardiovascular mortality risk [hazard ratio (HR): 0.92; 95%CI: 0.92, 0.93] and a 6% reduction in all-cause mortality risk (HR: 0.94; 95%CI: 0.94, 0.94). When eGDR was categorized, the negative correlation with cardiovascular and all-cause mortality progressively decreased from the lowest to the highest eGDR quartiles [Cardiovascular mortality: Q2 (HR: 1.01; 95%CI: 1.00, 1.01), Q3 (HR: 0.99; 95%CI: 0.98, 0.99), Q4 (HR: 0.78; 95%CI: 0.78, 0.78), *P*-trend <0.0001; All-cause mortality: Q2 (HR: 0.96; 95%CI: 0.96, 0.96), Q3 (HR: 1.00; 95%CI: 1.00, 1.00), Q4 (HR: 0.87; 95%CI: 0.87, 0.87), *P*-trend <0.0001].

**Table 2:** Multivariable Cox regression analysis of the relationship between eGDR and mortality.

	No. of case	HR (95%CI)				
		Non-adjusted Model	Model I	Model II	Model III	Model IV
Cardiovascular mortality						
eGDR		0.76 (0.76, 0.76)	0.88 (0.88, 0.88)	0.89 (0.89, 0.89)	0.91 (0.91, 0.91)	0.92 (0.92, 0.93)
eGDR (quartiles)						Ref
Q1	724 (6.29%)	Ref	Ref	Ref	Ref	
Q2	650 (5.65%)	0.73 (0.73, 0.73)	0.79 (0.79, 0.79)	0.94 (0.93, 0.94)	0.95 (0.95, 0.95)	1.01 (1.00, 1.01)
Q3	333 (2.90%)	0.32 (0.32, 0.32)	0.64 (0.64, 0.65)	0.82 (0.82, 0.83)	0.86 (0.86, 0.86)	0.99 (0.98, 0.99)
Q4	91 (0.79%)	0.09 (0.09, 0.09)	0.45 (0.45, 0.45)	0.64 (0.64, 0.65)	0.64 (0.63, 0.64)	0.78 (0.77, 0.78)
*P*-trend		<0.0001	<0.0001	<0.0001	<0.0001	<0.0001
All-cause mortality						
eGDR		0.80 (0.80, 0.81)	0.94 (0.94, 0.94)	0.92 (0.92, 0.92)	0.93 (0.93, 0.93)	0.94 (0.94, 0.94)
eGDR(quartiles)						
Q1	2526 (21.96%)	Ref	Ref	Ref	Ref	Ref
Q2	2372 (20.62%)	0.80 (0.80, 0.80)	0.85 (0.85, 0.85)	0.89 (0.89, 0.89)	0.93 (0.93, 0.93)	0.96 (0.96, 0.96)
Q3	1446 (12.58%)	0.44 (0.44, 0.45)	0.84 (0.84, 0.84)	0.89 (0.89, 0.89)	0.93 (0.93, 0.93)	1.00 (1.00, 1.00)
Q4	562 (4.88%)	0.16 (0.16, 0.17)	0.69 (0.69, 0.69)	0.75 (0.74, 0.75)	0.80 (0.80, 0.80)	0.87 (0.87, 0.87)
*P*-trend		<0.0001	<0.0001	<0.0001	<0.0001	<0.0001

HR, hazard ratios; CI, confidence interval; other abbreviations as in **Table 1**.

Model I adjusted for age, gender, race, PIR, level of education.

Model II adjusted for age, gender, race, PIR, level of education, height, BMI, SBP, DBP, ALT, AST, HDL-C, eGFR, HbA1c, UA.

Model III adjusted for age, gender, race, PIR, level of education, height, BMI, SBP, DBP, ALT, AST, HDL-C, eGFR, HbA1c, UA, drinking status, smoking status, congestive heart failure, coronary heart disease.

Model IV adjusted for age, gender, race, PIR, level of education, height, BMI, SBP, DBP, ALT, AST, HDL-C, eGFR, HbA1c, UA, drinking status, smoking status, congestive heart failure, coronary heart disease, antihypertensive therapy, hypoglycemic therapy, lipid-lowering therapy.

### Modifying effect of glycemic metabolic status

The modifying effect of glycemic metabolic status on the association between eGDR and cardiovascular and all-cause mortality was assessed. **Table 3** shows that glycemic metabolic status significantly modified the protective effects of high eGDR against cardiovascular and all-cause mortality risks (*P*-interaction<0.0001); specifically, high eGDR provided stronger protection against cardiovascular and all-cause mortality risks in individuals with NFG and prediabetes compared to those with diabetes.

**Table 3:** Multivariable Cox regression analysis of the modifying effect of glycemic metabolic status on the association of eGDR-related cardiovascular mortality and all-cause mortality.

	HR (95%CI)			P-interaction
	NFG	Prediabetes	Diabetes	
Cardiovascular mortality	0.89 (0.89, 0.89)	0.88 (0.88, 0.88)	0.95 (0.95, 0.95)	<0.0001
All-cause mortality	0.93 (0.93, 0.93)	0.95 (0.95, 0.95)	0.96 (0.96, 0.97)	<0.0001

HR, hazard ratios; CI, confidence interval; NFG, normal fasting glucose; other abbreviations as in **Table 1**.

Adjusted for age, gender, race, PIR, level of education, height, BMI, SBP, DBP, ALT, AST, HDL-C, eGFR, HbA1c, UA, drinking status, smoking status, congestive heart failure, coronary heart disease, antihypertensive therapy, hypoglycemic therapy, lipid-lowering therapy.

### Predictive value of eGDR in different glycemic metabolic states

**Figure 4:**
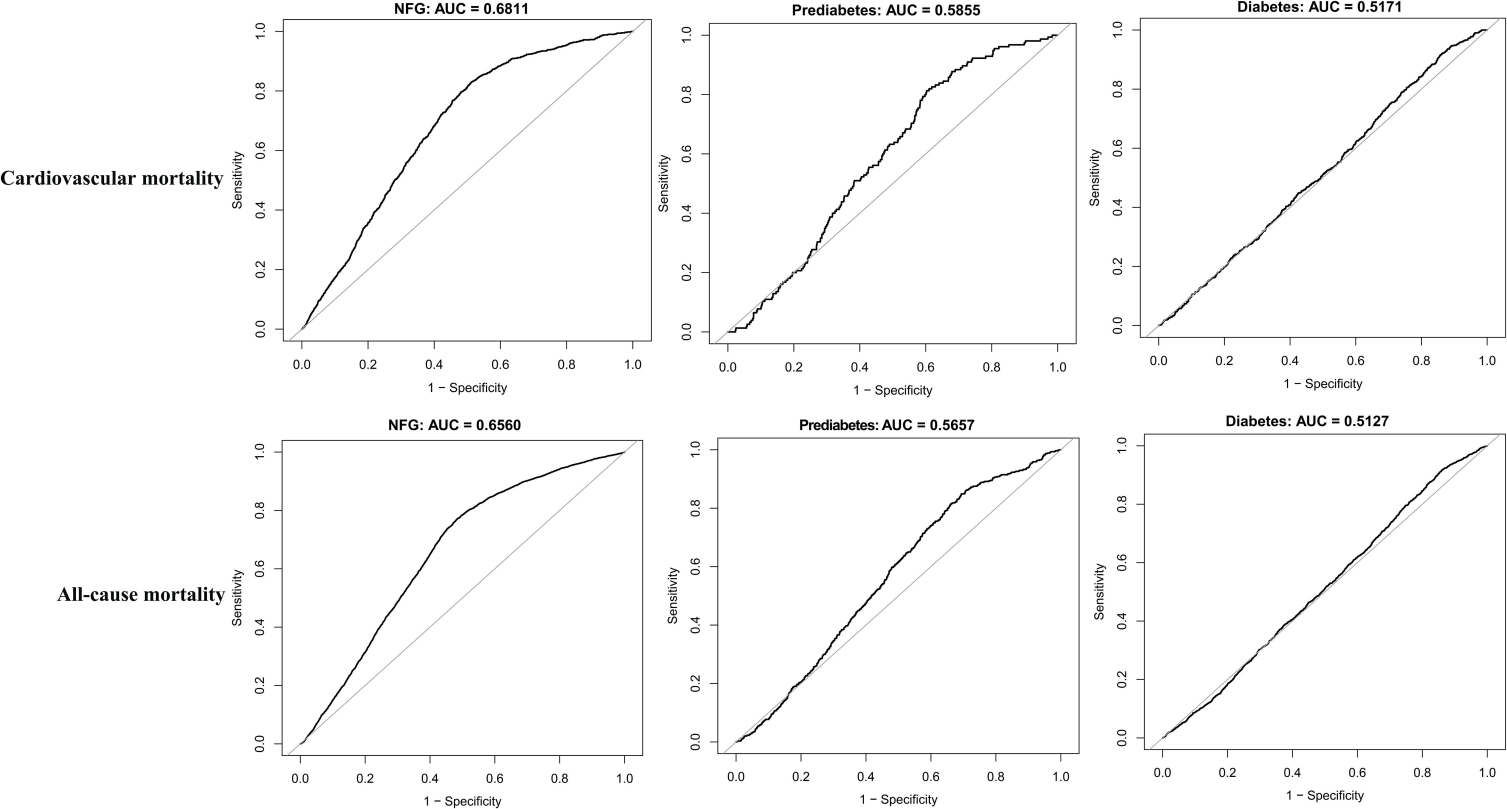
ROC curve analyzes the predictive value of eGDR for cardiovascular mortality and all-cause mortality under different glycemic metabolic statuses.

To further assess whether glycemic metabolic status influenced the predictive value of eGDR for cardiovascular and all-cause mortality, ROC curves were plotted, and AUC along with corresponding 95% CIs, optimal thresholds, sensitivity, and specificity were calculated (**Table 4**). The comparison of AUCs across different glycemic metabolic statuses revealed that eGDR had a higher predictive value for cardiovascular and all-cause mortality in individuals with NFG and prediabetes compared to those with diabetes (**Figure 4**); however, it should still be noted that in predicting death, the AUC of eGDR was less than 0.7 in all glycemic status groups.

**Table 4:** ROC analysis of the predictive value of eGDR for cardiovascular mortality and all-cause mortality under different glucose metabolism status.

	AUC	95%CI low	95%CI upp	Best threshold	Specificity	Sensitivity
Cardiovascular mortality						
NFG	0.6811	0.6671	0.6951	8.2625	0.4871	0.8306
Prediabetes	0.5855	0.5479	0.6231	6.9373	0.3974	0.8129
Diabetes	0.5171	0.4946	0.5395	7.2849	0.1425	0.9164
All-cause mortality						
NFG	0.6560	0.6480	0.6639	8.2149	0.5162	0.7708
Prediabetes	0.5657	0.5426	0.5888	8.2370	0.3065	0.8489
Diabetes	0.5127	0.4989	0.5265	7.5574	0.1395	0.9170

AUC, area under the curve; other abbreviations as in **Table 1**.

### Dose-response relationship between eGDR and mortality

**Figure 5:**
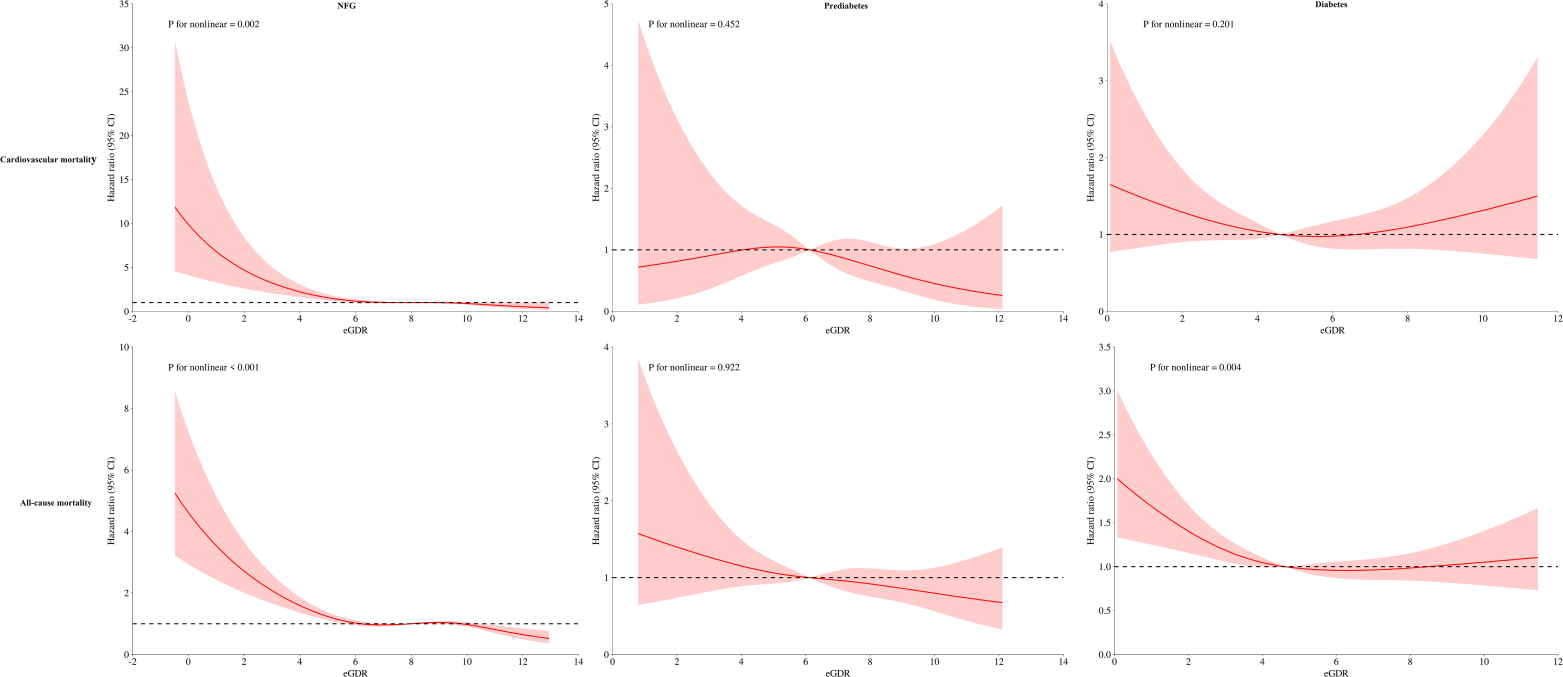
Dose-response relationship curves between eGDR and cardiovascular mortality and all-cause mortality under different glycemic metabolic statuses.

The dose-response relationship between eGDR and mortality was further analyzed using RCS. Results indicated a nonlinear association between eGDR and both cardiovascular and all-cause mortality in the NFG population; a similar nonlinear association was also observed for all-cause mortality in individuals with diabetes (**Figure 5**, *P* for nonlinearity<0.05). Segmental Cox regression identified eGDR thresholds for all-cause and cardiovascular mortality in the NFG population as 5.19 and 4.45, respectively, and 4.65 for all-cause mortality in the diabetes group (**Table 5**).

**Table 5:** The result of the two-piecewise Cox regression model.

	HR (95%CI)
Cardiovascular mortality (NFG group)	
The inflection point of eGDR	4.45
< 4.45	0.57 (0.46, 0.70)
> 4.45	0.93 (0.88, 0.99)
*P* for log likelihood ratio test	<0.001
All-cause mortality (NFG group)	
The inflection point of eGDR	5.19
< 5.19	0.73 (0.67, 0.80)
> 5.19	0.97 (0.94, 0.99)
*P* for log likelihood ratio test	<0.001
All-cause mortality (Diabetes group)	
The inflection point of eGDR	4.65
< 4.65	0.82 (0.76, 0.89)
> 4.65	1.03 (0.98, 1.08)
*P* for log likelihood ratio test	<0.001

HR, hazard ratios; CI, confidence; NFG, normal fasting glucose.

Adjusted for age, gender, race, PIR, level of education, height, BMI, SBP, DBP, ALT, AST, HDL-C, eGFR, HbA1c, UA, drinking status, smoking status, congestive heart failure, coronary heart disease, antihypertensive therapy, hypoglycemic therapy, lipid-lowering therapy.

## Discussion

Our study is one of the first to evaluate the impact of eGDR on cardiovascular and all-cause mortality risks across different glycemic metabolic statuses. The key findings can be summarized as follows: (1) Higher eGDR serves as a protective factor against both cardiovascular and all-cause mortality, with glycemic metabolic status significantly modulating this protective effect. Specifically, high eGDR offers stronger protective benefits against mortality risks in individuals with NFG and prediabetes compared to those with diabetes. (2) RCS analysis revealed a nonlinear relationship between eGDR and both cardiovascular and all-cause mortality in the NFG population; a similar nonlinear association was also noted in diabetic patients. (3) eGDR demonstrates greater accuracy in predicting cardiovascular and all-cause mortality in individuals with NFG and prediabetes than in those with diabetes.

IR is characterized by a decreased sensitivity and responsiveness to insulin, leading to impaired glucose utilization and subsequent metabolic abnormalities such as hyperglycemia (11). Numerous studies have established a close association between IR and the onset of cardiovascular diseases and strokes (11–13), as well as influencing the prognosis of various diseases (14–17). Our findings reinforce the notion that the surrogate marker for IR, eGDR, is closely linked to long-term cardiovascular and all-cause mortality. The biological mechanisms underlying the association between eGDR and mortality risks are not entirely clear, but insights can be gleaned from the adverse outcomes associated with IR: (1) Chronic IR can lead to a cascade of metabolic dysfunctions that cause damage to multiple organ systems, increasing the risks of cardiovascular and all-cause mortality (39); (2) The oxidative stress and inflammatory responses induced by IR can further elevate these risks (40); (3) IR can also lead to neuroendocrine dysregulation, such as the overactivation of the RAAS and sympathetic nervous systems, thereby increasing cardiac workload and impacting cardiovascular mortality risk (41).

As a surrogate for IR, eGDR integrates indices related to glucose, blood pressure, and obesity, providing a comprehensive assessment of IR (21). Previous research has demonstrated that low eGDR is associated with diabetic complications such as peripheral neuropathy (42), retinopathy (43), and progression of kidney diseases (44). Recent studies have further linked low eGDR with an increased risk of stroke and adverse cardiovascular and all-cause mortality events. For example, Zabala et al. found that a low eGDR significantly increased the risk of stroke and mortality in type 2 diabetes (25). Similar findings have been reported in populations with type 1 diabetes (T1DM), where low eGDR was associated with increased mortality (27), as well as in studies by Kong, Penno, and Olson (26, 28, 29). Our results corroborate these earlier findings, supporting the notion that low eGDR is a significant risk factor for cardiovascular and all-cause mortality.

In recent years, the focus of research has gradually shifted toward the impact of glycemic metabolic status. However, clinical understanding of different glycemic metabolic states remains limited due to a scarcity of studies (45, 46). From the perspective of time change trends, blood glucose metabolism status can be roughly divided into two categories: short-term changes and long-term status. In terms of short-term changes, the stress hyperglycemia ratio (SHR) may be a good parameter to study. Related studies have also reported some SHR-related research results (47, 48); For example, Lai et al. examined the association between SHR and adverse chronic kidney disease outcomes in adults with diabetes in the United States. They found that either too low or too high SHR levels were significantly associated with adverse renal outcomes in the diabetic population (47). A similar finding was reported in a recent study by Tian et al., who reported a U-shaped association of SHR with mortality in patients with cardiogenic shock (48). Additionally, hyperglycemia itself can have adverse effects on the body, it can be debilitating in older people (49), and can affect normal fertility in women of childbearing age (50). This may be related to the inflammatory reaction or hormonal changes secondary to hyperglycemia, which in turn affects the thickness of the endometrium and causes infertility (50), and even affects the therapeutic effect of intrauterine insemination (51). For the long-term status of blood glucose metabolism, this requires comprehensive assessment and diagnosis based on relatively comprehensive blood glucose measurement information to determine the classification of the blood glucose status of the study population: the main categories are normoglycemic state, prediabetic state and diabetic state. In general, studies on glucose metabolism status that have been completed in the past have focused on metabolism-related diseases, such as the impact of glycemic control on carotid atherosclerosis plaque (52, 53) and CHD (54, 55). Furthermore, Wu et al. found that glycemic metabolic status could modulate the influence of atherosclerotic indices on CHD (56), while Zhang et al. discovered that the SHR was significantly related to the risk and predicted severity of multi-vessel CHD, especially in individuals with prediabetes and diabetes (57). These findings underscore the potential clinical significance of incorporating glycemic metabolic status into practice.

Our study further explores the association between eGDR and mortality risks across different glycemic statuses, finding a more pronounced negative correlation between eGDR and mortality risks in NFG and prediabetes compared to diabetes. This suggests that, at the same high level of eGDR, the mortality risk reduction benefits are relatively smaller for diabetic patients. This may be due to the higher complication risks associated with diabetes, which increase cardiovascular and overall mortality risks (58–60). Additionally, diabetes, as a strong exposure risk factor, increases the risks of cardiovascular and all-cause mortality (9, 61). Furthermore, it should be noted that in the current study, we also found that eGDR was negatively correlated with both prediabetes and diabetes risk; in contrast, high levels of eGDR may have a weaker protective effect on diabetes. In view of this finding, we consider that the weak protective effect of high eGDR on diabetes may further persist into the poor prognosis of diabetic patients. Based on the above correlation analysis, the current study also assesses the predictive value of eGDR for these risks, showing more accurate predictions in NFG and prediabetes than in diabetes. The integration of correlation analysis and ROC findings suggests that high eGDR provides enhanced protection and predictive value for populations with preferred glycemic metabolism.

Further RCS analysis revealed interesting results: eGDR demonstrated a nonlinear, L-shaped relationship with mortality risks in the NFG (for cardiovascular and all-cause mortality) and diabetic (for all-cause mortality) populations. The RCS model indicated that as eGDR levels incrementally increase, the risks of cardiovascular and all-cause mortality progressively decreased, stabilizing once eGDR surpassed a certain threshold. Similar L-shaped relationships were previously reported in a study on a Swiss T1DM population by Nyström et al., where the threshold for eGDR was identified to be between 8-10, beyond which all-cause mortality risks stabilized (27). Although the threshold values from Nyström et al.’s study slightly differ from ours, likely due to population and disease type differences, the overall trends align with our findings, underscoring the nuanced interactions between eGDR levels and health outcomes.

The findings of the current study have implications for daily clinical practice. Given the NHANES sampling and weighting methods, the findings are highly applicable to the general population. From the perspective of eGDR development background, eGDR is obtained through a comprehensive assessment of hypertension, HbA1c and obesity. It is easy and convenient to obtain and can quickly understand the IR status of the population. Therefore, the clinical significance of the current study is to offer a simple surrogate for IR to predict poor prognosis across various glycemic statuses. In addition, the evidence from the current study can also provide useful research materials for subsequent further studies. Considering the limited value of eGDR for mortality prediction in the population of all glycemic states in the current study, we suggest incorporating eGDR as an add-on in future studies to develop more accurate prediction models.

### Strengths and limitations

There are several strengths of the current study worth mentioning: (1) This study is the first to explore the effects of eGDR on cardiovascular and all-cause mortality risks across different glycemic metabolic statuses. (2) The NHANES national database, designed through a complex multistage probability sampling and controlling for potential confounders in multivariable models, lends reliability to the findings.

This article still has some shortcomings: (1) This study was conducted solely within the U.S. population, and further research is needed to determine whether the results can be generalized to other populations. (2) The endpoint events were obtained from NCHS, and there might be a delay in the recording of some mortality cases, potentially leading to an underestimation of actual mortality risks. (3) This was not a predefined analysis; such observational studies are susceptible to potential biases and confounding factors. (4) Some data were inevitably missing from the surveys, leading to the exclusion of some samples from the analysis, which could introduce selection bias. Furthermore, to ensure a viable sample size for analysis, variables with a high level of missing data such as TG, LDL-C, and FPG (over 50%) were excluded from the multivariable models [see **Supplementary Table S6** for missing information], which may introduce some selection bias. However, the larger sample size used in the analyses helps to reinforce and support the study results.

## Conclusion

In the U.S. adult population, high eGDR acts as a protective factor against cardiovascular and all-cause mortality, with glycemic metabolic status significantly modifying this protective effect. Specifically, high eGDR provides stronger protection for individuals with NFG and prediabetes. It is also noteworthy that eGDR appears to be more applicable for predicting mortality in populations with NFG and prediabetes, suggesting its potential utility in clinical settings for these groups. Future research should aim to explore these relationships in diverse populations and settings to validate and extend these findings.

## Data Availability

The raw data supporting the conclusions of this article will be made available by the authors, without undue reservation.
