# Supplementary material for: Analyzing the impact of glycemic metabolic status on cardiovascular mortality and all-cause mortality related to the estimated glucose disposal rate: a nationwide cohort study
Source: Front Endocrinol (Lausanne). 2025 Jan 21;15:1494820. doi: 10.3389/fendo.2024.1494820 (PMC11790456; doi:10.3389/fendo.2024.1494820)
Supplement: Supplementary file 1 [file Table1.docx]

Supplementary Table 1: Diagnostic steps for collinearity between eGDR and other covariates when cardiovascular mortality as the dependent variable.

|  | VIF | | | | |
| --- | --- | --- | --- | --- | --- |
|  | Step 1 | Step 2 | Step 3 | Step 4 | Step 5 |
| eGDR | Inf | 36.8 | 36.8 | 35.2 | 4.6 |
| Age | 3.6 | 3.6 | 3.6 | 3.6 | 3.3 |
| Gender | 2.5 | 2.5 | 2.5 | 2.5 | 2.5 |
| Race | 1.1 | 1.1 | 1.1 | 1.1 | 1.1 |
| PIR | 1.3 | 1.3 | 1.3 | 1.3 | 1.3 |
| Education | 1.3 | 1.3 | 1.3 | 1.3 | 1.3 |
| Drinking status | 1.2 | 1.2 | 1.2 | 1.2 | 1.2 |
| Smoking status | 1.1 | 1.1 | 1.1 | 1.1 | 1.1 |
| Weight | 91.8 | 91.8 | 91.8 | NA | NA |
| Height | 22.2 | 22.2 | 22.2 | 2.8 | 2.3 |
| BMI | 71.2 | 71.2 | 71.2 | 8.5 | 2.5 |
| WC | Inf | NA | NA | NA | NA |
| SBP | 1.8 | 1.8 | 1.8 | 1.8 | 1.8 |
| DBP | 1.4 | 1.4 | 1.4 | 1.4 | 1.4 |
| HDL-C | 2306.8 | 2306.8 | 1.5 | 1.5 | 1.5 |
| LDL-C | 11741.4 | 11741.4 | 1.2 | 1.2 | 1.2 |
| TC | 15206.5 | 15206.5 | NA | NA | NA |
| TG | 1682 | 1682 | 1.4 | 1.4 | 1.4 |
| ALT | 2.2 | 2.2 | 2.2 | 2.2 | 2.2 |
| AST | 2.2 | 2.2 | 2.2 | 2.2 | 2.2 |
| eGFR | 2.5 | 2.5 | 2.5 | 2.5 | 2.5 |
| HbA1c | Inf | 5.1 | 5.1 | 5.1 | 3.9 |
| FPG | 3.3 | 3.3 | 3.3 | 3.3 | 3.3 |
| UA | 1.6 | 1.6 | 1.6 | 1.6 | 1.6 |
| Hypertension | Inf | 16.3 | 16.3 | 15.7 | NA |
| Congestive heart failure | 1.2 | 1.2 | 1.2 | 1.2 | 1.2 |
| Coronary heart disease | 1.2 | 1.2 | 1.2 | 1.2 | 1.2 |
| Antihypertensive therapy | 2 | 2 | 2 | 2 | 1.9 |
| Hypoglycemic therapy | 1.6 | 1.6 | 1.6 | 1.6 | 1.6 |
| Lipid-lowering therapy | 1.7 | 1.7 | 1.7 | 1.7 | 1.7 |

VIF: variance inflation factor; VIF = 1/(1-R^2^). Abbreviations as in Table ​1.

Note: The variables with VIF>10 will be regarded as collinear variables and cannot be included in the multiple regression model.

Supplementary Table 2: Diagnostic steps for collinearity between eGDR and other covariates when all cause mortality as the dependent variable.

|  | VIF | | | | |
| --- | --- | --- | --- | --- | --- |
|  | Step 1 | Step 2 | Step 3 | Step 4 | Step 5 |
| eGDR | Inf | 36.8 | 36.8 | 35.2 | 4.6 |
| Age | 3.6 | 3.6 | 3.6 | 3.6 | 3.3 |
| Gender | 2.5 | 2.5 | 2.5 | 2.5 | 2.5 |
| Race | 1.1 | 1.1 | 1.1 | 1.1 | 1.1 |
| PIR | 1.3 | 1.3 | 1.3 | 1.3 | 1.3 |
| Education | 1.3 | 1.3 | 1.3 | 1.3 | 1.3 |
| Drinking status | 1.2 | 1.2 | 1.2 | 1.2 | 1.2 |
| Smoking status | 1.1 | 1.1 | 1.1 | 1.1 | 1.1 |
| Weight | 91.8 | 91.8 | 91.8 | NA | NA |
| Height | 22.2 | 22.2 | 22.2 | 2.8 | 2.3 |
| BMI | 71.2 | 71.2 | 71.2 | 8.5 | 2.5 |
| WC | Inf | NA | NA | NA | NA |
| SBP | 1.8 | 1.8 | 1.8 | 1.8 | 1.8 |
| DBP | 1.4 | 1.4 | 1.4 | 1.4 | 1.4 |
| HDL-C | 2306.8 | 2306.8 | 1.5 | 1.5 | 1.5 |
| LDL-C | 11741.4 | 11741.4 | 1.2 | 1.2 | 1.2 |
| TC | 15206.5 | 15206.5 | NA | NA | NA |
| TG | 1682 | 1682 | 1.4 | 1.4 | 1.4 |
| ALT | 2.2 | 2.2 | 2.2 | 2.2 | 2.2 |
| AST | 2.2 | 2.2 | 2.2 | 2.2 | 2.2 |
| eGFR | 2.5 | 2.5 | 2.5 | 2.5 | 2.5 |
| HbA1c | Inf | 5.1 | 5.1 | 5.1 | 3.9 |
| FPG | 3.3 | 3.3 | 3.3 | 3.3 | 3.3 |
| UA | 1.6 | 1.6 | 1.6 | 1.6 | 1.6 |
| Hypertension | Inf | 16.3 | 16.3 | 15.7 | NA |
| Congestive heart failure | 1.2 | 1.2 | 1.2 | 1.2 | 1.2 |
| Coronary heart disease | 1.2 | 1.2 | 1.2 | 1.2 | 1.2 |
| Antihypertensive therapy | 2 | 2 | 2 | 2 | 1.9 |
| Hypoglycemic therapy | 1.6 | 1.6 | 1.6 | 1.6 | 1.6 |
| Lipid-lowering therapy | 1.7 | 1.7 | 1.7 | 1.7 | 1.7 |

VIF: variance inflation factor; VIF = 1/(1-R^2^). Abbreviations as in Table ​1.

Note: The variables with VIF>10 will be regarded as collinear variables and cannot be included in the multiple regression model.

Supplementary Table 3: Mult-class logistic regression analysis of the association between eGDR and prediabetes and diabetes.

|  | OR (95% CI) | | | |
| --- | --- | --- | --- | --- |
|  | Model I | Model II | Model III | Model IV |
| NFG | Ref | Ref | Ref | Ref |
| Prediabetes | 0.58 (0.58, 0.58) | 0.80 (0.80, 0.80) | 0.79(0.79, 0.80) | 0.88 (0.88, 0.88) |
| Diabetes | 0.83 (0.83, 0.83) | 0.91 (0.91, 0.91) | 0.91 (0.90, 0.91) | 0.93 (0.93, 0.93) |

Abbreviations: OR: Odds ratios; CI: confidence interval; other abbreviations as in Table 1.

Model I adjusted for age, gender, race, PIR, level of education;

Model II adjusted for age, gender, race, PIR, level of education, height, BMI, SBP, DBP, ALT, AST, HDL-C, eGFR, HbA1c, UA.

Model III adjusted for age, gender, race, PIR, level of education, height, BMI, SBP, DBP, ALT, AST, HDL-C, eGFR, HbA1c, UA, drinking status, smoking status, congestive heart failure, coronary heart disease.

Model IV adjusted for age, gender, race, PIR, level of education, height, BMI, SBP, DBP, ALT, AST, HDL-C, eGFR, HbA1c, UA, drinking status, smoking status, congestive heart failure, coronary heart disease, antihypertensive therapy, hypoglycemic therapy, lipid-lowering therapy.

Supplementary Table 4: Mortality rates corresponding to eGDR quartile groups.

|  | eGDR quartiles | | | |
| --- | --- | --- | --- | --- |
|  | Q1 | Q2 | Q3 | Q4 |
| Cardiovascular mortality | 2526 (21.96%) | 2372 (20.62%) | 1446 (12.58%) | 562 (4.88%) |
| All-cause mortality | 724 (6.29%) | 650 (5.65%) | 333 (2.90%) | 91 (0.79%) |

Abbreviations: eGDR: estimated glucose disposal rate.

Supplementary Table 5: Mortality rates corresponding to different glycemic metabolic statuses.

|  | glycemic metabolic statuses | | | |
| --- | --- | --- | --- | --- |
|  | NFG | Prediabetes |  | Diabetes |
| Cardiovascular mortality | 4128 (11.92%) | 609 (18.11%) |  | 2169 (27.00%) |
| All-cause mortality | 1021 (2.95%) | 155 (4.61%) |  | 622 (7.74%) |

Abbreviations: NFG: normal fasting glucose.

Supplemental Table 6: The missing number and rate of covariates.

|  | Non- Missing | Missing |
| --- | --- | --- |
| Age | 46016 | 0 |
| Gender | 46016 | 0 |
| Race | 46016 | 0 |
| PIR | 42108 | 3908 |
| Education | 45963 | 53 |
| Drinking status | 42149 | 3867 |
| Smoking status | 45979 | 37 |
| Weight | 45944 | 72 |
| Height | 45883 | 133 |
| BMI | 45823 | 193 |
| WC | 46016 | 0 |
| SBP | 44685 | 1331 |
| DBP | 44487 | 1529 |
| HDL-C | 45431 | 585 |
| LDL-C | 21215 | 24801 |
| TC | 45433 | 583 |
| TG | 21990 | 24026 |
| ALT | 45232 | 784 |
| AST | 45215 | 801 |
| eGFR | 45314 | 702 |
| HbA1c | 46016 | 0 |
| FPG | 22346 | 23670 |
| UA | 45302 | 714 |
| Hypertension | 46016 | 0 |
| Congestive heart failure | 45882 | 134 |
| Coronary heart disease | 45820 | 196 |
| Antihypertensive therapy | 45981 | 35 |
| Hypoglycemic therapy | 45981 | 35 |
| Lipid-lowering therapy | 45981 | 35 |

Abbreviations as in table 1.
